# Effect of soil fumigants on degradation of abamectin and their combination synergistic effect to root-knot nematode

**DOI:** 10.1371/journal.pone.0188245

**Published:** 2018-06-11

**Authors:** Bin Huang, Jun Li, Qian Wang, Meixia Guo, Dongdong Yan, Wensheng Fang, Zongjie Ren, Qiuxia Wang, Canbin Ouyang, Yuan Li, Aocheng Cao

**Affiliations:** 1 Institute of Plant Protection, Chinese Academy of Agricultural Sciences, Beijing, China; 2 State Key Laboratory for Biology of Plant Disease and Insect Pests, Beijing, China; Institute for Sustainable Plant Protection, C.N.R., ITALY

## Abstract

**Background:**

Root-knot nematode (*Meloidogyne spp*., RKN*)* causes a disease that significantly reduces the yield of greenhouse cucumber crops year after year. Chemical control based on a single pesticide is now unreliable mainly due to pest resistance. Fumigant and non-fumigant pesticide combinations can potentially result in effective and economic RKN control.

**Results:**

Combining the insecticide abamectin (ABM) with fumigants dazomet (DZ) or chloropicrin (CP) significantly extended the half-life of ABM by an average of about 1.68 and 1.56 times respectively in laboratory trials, and by an average of about 2.02 and 1.69 times respectively in greenhouse trials. Laboratory experiments indicated that all the low rate ABM combination treatments controlled RKN through a synergistic effect. ABM diffused into the nematode epidermis more rapidly when ABM was combined with DZ and CP, giving effective nematode control and an increase cucumber total yield, compared to the use of these products alone. ABM combined with CP or DZ produced significantly higher total cucumber yield than when these products were used alone.

**Conclusions:**

A low concentration of ABM combined with DZ in preference to CP would be an economic and practical way to control nematode and soilborne fungi in a greenhouse producing cucumbers.

## Introduction

Continually planting cucumber, tomato and pepper crops in greenhouses year after year without soil treatments eventually leads to an increase in soilborne pathogens and plant disease, which reduce crop yield and quality [1]. Soilborne diseases caused by Fusarium wilt and Root Knot Nematode (*Meloidogyne spp*., RKN) can be carried over each year by diseased plants, soil and seeds [2]. Soil fumigation is the most effective and reliable way to prevent soilborne diseases being perpetuated in greenhouse crop production [3,4]. Methyl bromide (MB) was the most commonly used fumigant until it was phased out globally under the Montreal Protocol due to its ability to damage the ozone layer [5]. It has been replaced by chloropicrin (CP) and dazomet (DZ) which are now commonly-used fumigants worldwide for cucumber production [6–10].

Although CP and DZ provide effective control of soilborne fungi and bacteria comparable to MB [9,11–14], they provide only moderate control of nematodes [4,15]. Increased use of CP and DZ in several glasshouse-produced crops has led to RKN disease becoming more prevalent. RKN penetrates the roots of crops, thereby facilitating further infection of the plant by soilborne pathogens, which reduces crop yield by 15% to 40% or more in some cases [16]. Growers have relied mainly on organophosphate and carbamate insecticides to control diseases caused by RKN [17,18], which has led to enhanced biological degradation of nematicides and prevalence of pathogens. Under these circumstances, increasing the nematicide dose only increases cost without improving pathogen control. It has become paramount to find a combination of fumigant and non-fumigant pesticides as soon as possible to efficiently control RKN and key soilborne fungi.

Abamectin (5-O-demethylavermectin A1a (i) mixture with 5-O-demethyl-25-de (1-methylpropyl)-25-(1-methylethyl)avermectin) is highly toxic nematicide commonly-used to control RKN in China [18,19]. Abamectin (ABM) is produced commercially as a natural fermentation product by the soil bacterium *Streptomyces avermitilis*. Farmers apply ABM to control RKN mainly by using root-irrigation and hole fertilization methods. However, these methods require relatively large quantities of ABM which were reported to damage the plant roots of some crops [20,21]. Importantly, RKN populations must be controlled before RKN enters the roots of the host because once inside it is more difficult for pesticides to make direct contact with the pest [17,22]. As ABM is reported to be degraded by sunlight, increased temperature and high soil humidity [23,24], its ability to come into contact with RKN is reduced under these conditions which are characteristic of most greenhouses. Soil fumigation is known to kill most of the soil microorganism communities and non-target organisms [25, 26], to increase nutrient mineralization rate and to also increase soil fertility and crop yield [11,27]. However, relatively little is known about the effect of fumigants such as CP and DZ on ABM degradation rate and the period of time after application of ABM that still provides effective RKN control, particularly when ABM is combined with CP and DZ.

The present work aimed to determine the ability of CP, DZ and ABM combinations to control RKN and to increase cucumber yield; to determine the effect of DZ and CP on the degradation of ABM in laboratory and greenhouse trials; and to explore whether DZ and CP can facilitate and even enhance ABM’s ability to diffuse across the nematode’s epidermis and thereby improve control of this pest. The results of this study will be used to guide the effective, economic and practical use of ABM in combination with CP and DK to control plant diseases caused by RKN and any soilborne fungi that may be present incidentally. The results may also lead to an improved understanding of the synergistic mechanisms that significantly improves the efficacy of the fumigant (CP, DZ) and non-fumigant (ABM) combination.

## Materials and methods

### Chemicals, reagents and soil

The sources of chemicals, reagents, purity and supplier are described in Table 1. A standard stock solution of ABM (100 mg L^-1^) was prepared in pure acetonitrile. Standard working solutions at concentrations of 0.01, 0.05, 0.1, 0.2, 0.5 and 1 mg L^-1^ were prepared from the stock solution by serial dilution in acetonitrile (ACN). All the solutions were stored in aluminum foil in a refrigerator in the dark at -20°C prior to analysis. The working standard solutions remained stable for three months.

**Table 1:** Sources of chemicals and reagents.

Chemical	Abbreviation	Purity	Supplier
Chloropicrin Technical (TC)	CP	> 99.5%	Dalian Lvfeng Chemical Co. Ltd (Dalian, China)
Dazomet microgranule (MG)	DZ	>98%	Nantong Shizhuang Chemical Co. Ltd., China
Abamectin Standard	ABM	94.2%	AccuStandard Inc., (New Haven, USA)
Abamectin emulsifiable concentrate (EC)	ABM-EC	5%	Shenzhen Noposion Agrochemicals Co. Ltd (Shenzhen, China)
HPLC-grade acetonitrile	ACN	HPLC-grade	Tedia (Fairfield, OH, USA)
HPLC-grade formic acid		HPLC-grade	Tedia (Fairfield, OH, USA)
Analytical grade acetonitrile, anhydrous magnesium sulfate and sodium chloride	MgSO_4_ NaCl	Analytical grade	Sinopharm Chemical Reagent Co. Ltd (Beijing, China)
Cleanert Florisil		Analytical grade	Bonna-Agela Technologies Inc. (Tianjin, China)
Primary secondary amine and nylon syringe filters (0.22μm)	PSA	Analytical grade	Agela Technologies (Newark, USA)

The soil used in the laboratory study for Trial 1 was collected from within 30cm of the surface of unfumigated areas a greenhouse located in Shunyi (Beijing). This glasshouse had grown cucumber for more than three years and had reported a significant RKN disease incidence. The soil used in Trial 2 was from a nearby glasshouse also located in Shunyi. The main physicochemical characteristics of the soils used in the laboratory and greenhouse trials are described in Table 2 [28,29].

**Table 2:** The main physicochemical characteristics of soil collected in laboratory and greenhouse trials.

Source of soil	Sand (%)	Silt (%)	Clay (%)	NH_4_^+^ -N (mg kg^−1^)	NO_3_^-^-N (mg kg^−1^)	Available phosphorus (mg kg^−1^)	Organic matter (g kg^−1^)	pH	Water content (%)
Trial 1	12.3	64.4	23.3	35.7	56.2	315.6	33.5	6.5	17.8
Trial 2	16.7	70.5	12.8	12.5	23.8	172.6	26.1	6.9	23.6

### Laboratory tests

#### Degradation of nematicides in the laboratory

The soil collected from the cucumber greenhouses was sieved through a 2 mm screen and then incubated in glass desiccators for 7 days at 25°C. To determine the effect of CP or DZ on the degradation of ABM in soil, the soil moisture was adjusted to 30% maximum field capacity (Water content: 14.3%).

The doses of ABM treatments in each desiccator were 0, 0.01, 0.05, 0.1, 0.5, 1, 2, 4, 8, 16 mg kg^-1^. The LC_50_ of ABM against RKN was determined in a pre-test to be 0.75 mg kg^-1^, which provided guidance on the range of ABM rates likely to be of interest in these experiments. About 300 g of *Meloidogyne*-infested soil was mixed evenly into in each desiccator. The desiccators were then incubated at 25°C for 5 days.

A repeat, two-factor completely randomised design was carried out in these laboratory experiments. The fumigant factor consisted of two fumigant treatments: CP (Recommended rate: 40mg kg^−1^) and DZ (Recommended rate: 100mg kg^−1^) and an untreated control (CK) [9,11]. The second factor ABM had two doses: A high dose of 1.50mg kg^−1^, and a low dose of 0.75mg kg^−1^. There were six combinations of treatment levels. Eighteen 2.0L desiccators each containing 800g soil were selected and randomly divided into 6 groups. Each group received a combination of treatment factors and each combination of treatment factors was replicated three times. Standard solutions of ABM were evenly mixed into the soil in each desiccator and the soil water content was adjusted to 30% of the maximum field capacity. CP was injected into the soil and DZ was sprinkled. The desiccator lids were sealed to the bases with a coating of vaseline before being incubated at 25°C for 7 days. After 7 days of fumigation, the desiccators were moved into a ventilation hood to vent the fumigation gases. After initial sampling, the remainder of the treated soils were transferred to a desiccator and incubated at 25°C. The moisture content was adjusted to 30% of the maximum field capacity every five days. Soil samples were taken at 2 h, then 7, 14, 21, 28, 35, 42, 60 and 90 d after ABM had been added. Fumigants CP (Recommended rate: 40mg kg^−1^, Low rate: 20mg kg^−1^) and DZ (Recommended rate: 100mg kg^−1^, Low rate: 50mg kg^−1^) were combined with ABM and the levels of nematode and pathogen control were recorded 7 days after ABM application.

In all the experiments, RKN (*Meloidogyne spp*.) was isolated from the soil and quantified using methods described by Liu [30]. *Fusarium spp*. and *Phytophthora spp*. were isolated and quantified using methods described by Komada [31] and Yates *et al*. [32], respectively. All the solutions were protected against light with aluminum foil and stored in a refrigerator at—20°C until analyzed.

#### Measurement of abamectin penetration through the epidermis of the root-knot nematode when combined with CP and DZ

CP, DZ and ABM were formulated as aqueous solutions at nearly twice the concentration reported to result in an LC_50_ for RKN in water. One ml of 2000 root-knot nematode/ml suspension or pure water was transferred to each 10ml centrifuge tube. The 1 ml of pure water was used as a blank control. One ml of CP, DZ or ABM aqueous solution was then transferred to a 10ml centrifuge tube which was then sealed. Samples were taken from the tubes after 1, 2, 4, 6, 8, 10 and 24 h incubation at 25°C. The samples were screened through a 400 mesh sieve. One ml of ACN was pipetted and used to leach down the ABM from the nematode epidermis from the sieve three times. ABM eluent was collected and 1ml of it was transferred to a 1.5ml vial to be quantified. The 1 ml eluent of CP or DZ was extracted three times with ethyl acetate and 1ml extracted each time. The upper organic phase was then collected for analysis by gas chromatography [14,33].

### Greenhouse trials

#### Degradation of nematicides in greenhouse trials

Two trials on soils from greenhouses were carried out in 2016 and 2017. The greenhouses in Shunyi (Beijing) had produced cucumber (cultivar: No. 16 Zhongnong) for more than 5 years and were reported to be heavily infected with RKN (*Meloidogyne spp*.) and soilborne fungi (*Fusarium spp*. and *Phytophthora spp*.).

A repeat, two-factor completely random design was carried out on the greenhouse soils. The fumigant factor in the two greenhouse trials consisted of two fumigant treatments (CP, DZ) and an unfumigated control (CK). The ABM rate as the second factor varied from a high of 0.75gm^−2^ to a low rate of 0.375gm^−2^ as the emulsifiable concentrate (ABM-EC) formulation. Eighteen similar plots were selected in each greenhouse trials and randomly divided into 6 groups. Each group received a combination of treatment levels. Each plot (3.2m×5.8m) had 4 rows of 60 cucumbers replicated three times. The treatments, plot areas, application method and pesticide rates in Trials 1 and 2 are summarized in Table 3. After application of the ABM-EC by a sprayer, DZ was applied by mixing it with soil at a rate of 30 gm^−2^, and then rotary tilled. CP was applied at the chisel injection rate of 20 gm^−2^ after application of the ABM-EC and rotary tilled. All the treatments were covered with polyethylene film (thickness 0.04 mm). Ten days after fumigation, the film was removed to vent the fumigant gas. Cucumber seedlings were planted about 10 days later. Soil samples were collected at 1, 3, 5, 7, 10, 14, 21, 35, 60, 90 d after ABM application. Soil was sampled from at least five locations in each plot. Soil sample weight from in each location was recorded and always exceeded 300g. All soil samples were refrigerated -20°C before analysis.

**Table 3:** Experimental treatments in the greenhouse trials.

Trial site	Soil fumigant	Fumigant dose (gm^-2^)	Abamectin	Nematicide rate (gm^-2^)	Plot area (m^2^)	Tarp type	Application method
Trial 1 and Trial 2	CP	20	high rate	0.75	18.56	PE	Spray+chisel injection
			low rate	0.375	18.56	PE	Spray+chisel injection
				—	18.56	PE	Chisel injection
	DZ	30	high rate	0.75	18.56	PE	Spray+soil mixture
			low rate	0.375	18.56	PE	Spray+soil mixture
				—	18.56	PE	Soil mixture
	CK	—	high rate	0.75	18.56	PE	Spray
			low rate	0.375	18.56	PE	Spray
	BG	—	—	—	18.56	—	—

#### Effect of the treatments on the control of root-knot nematode, soilborne fungi and cucumber production

To determine the control of the treatments on RKN and soilborne fungi, samples of soil were collected before the ABM was applied, and then at 7, 21, 35, 60 and 90 d after the ABM was initially applied to the soil. The height of the cucumber plants in the two trials was measured on the 90th day. The yield of the cucumber and the cucumber sale price was recorded every day from the first to the last picking day.

### Extraction and analysis of samples

#### Sample extraction

A 10g finely-homogenised soil sample was weighed into a 50 ml Teflon centrifuge tube. Five ml of water and 10 ml MeCN were added. A recovery study was performed by spiking each matrix with the ABM standard solutions described above. The mixtures were shaken vigorously for 3 min by a shaker and sonicated for 15 min in an ultrasonic bath. Subsequently, 4 g anhydrous MgSO_4_ and 1 g NaCl were added to the mixture. The tubes were shaken for 3 min and centrifuged at a relative centrifugal force 2811×g for 5 min. A 2 ml aliquot was transferred into a 10 ml centrifuge tube containing 50 mg Florisil and 200 mg anhydrous MgSO_4_. The tube was vortexed for 30 s and was again centrifuged at a relative centrifugal force 2811×g for 5 min. The supernatant was filtered through a 0.22 μm nylon syringe filter into a 1.5 ml vial for UHPLC−MS/MS injection [34].

#### UPLC–MS/MS analysis

The analysis of ABM was carried out on Waters Acquity UPLC system coupled to a triple-quadrupole mass spectrometer equipped with an ESI source (Waters Corporation USA). Chromatographic separation was performed using an Acquity UPLC BEH C18 1.7 μm column (2.7×100 mm) (Waters Corporation USA). The mobile phases, which were composed of MeCN (A) and 0.2% v/v formic acid in water (B), were pumped at a flow rate of 0.3 ml/min. The gradient program was as follows: 0 min, 10% A; 1.5 min, 90% A; 2.5 min, 90% A; 2.6 min, 10% A; 5 min, 10% A. The injection volume was 3 μl and the column was kept at 40°C. The ABM was eluted for 2.32 min. The multiple reaction monitoring mode was used in the positive-ion mode. The parent ion of ABM was 895.46 m/z and the quantification ion was 183.04 m/z, and the confirmatory ion was 327.09 m/z. The cone voltages and collision energies of the quantification ion and confirmatory ion were 2 V and 54 V, respectively [35].

### Data analyses

Previous studies showed that ABM degradation followed a single first-order model [21,24]. The degradation of ABM was therefore calculated in these experiments using the following equation:

$$C_{\left(t\right)}=C_{0}\times e^{-kt}$$

where t is the time after application of ABM; k is the 1st-order rate constant; C(t) is the concentration of the residue at time t in soil and C_0_ is the initial concentration of ABM. Half-life values were calculated based on this 1st-order model.

The diffusion rate of ABM through the epidermis of RKN was calculated by the following equation [36]:

$$X\%=\left(1-\frac{C_{X}}{C_{0}}\right)\times 100\%$$

where X% is the epidermal diffusion rate; C_0_ is the initial concentration of ABM before put it in an incubator; C_x_ is the concentration of ABM after leaching from the epidermis of RKN.

Theoretical control efficacy of fumigant DZ or CP in combination with ABM was calculated by the following equation [37]:

$$E_{0}=X+Y\times \frac{100-X}{100}$$

where E_0_ is the theoretical control efficacy of fumigant combined with ABM (%); X is the single control efficacy of ABM (%); Y is the single control efficacy of fumigant DZ or CP (%); E is the observed control efficacy of fumigant combination with ABM (%). If E- E_0_ > 0, the combination was synergistic; if E- E_0_ < 0, the combination was antagonistic.

Nematode mortality was calculated using the following equation:

$$X=\frac{N_{1}}{\left(N_{1}+N_{2}\right)}\times 100$$

where X is the nematode mortality (%); N_1_ is the number of dead nematodes; and N_2_ is the number of surviving nematodes.

The efficacy of RKN (corrected mortality rate) can be described by the following equation:

$$Y=\frac{X_{1}-X_{2}}{1-X_{1}}\times 100$$

where Y is the corrected nematode mortality (%); X_1_ is the nematode mortality of fumigant or ABM treatment (%); X_2_ is the blank control mortality (%).

The efficacy of soilborne fungi (inhibition rate) was calculated by the following equation:

$$Y=\frac{N_{1}-N_{2}}{N_{2}}\times 100$$

where Y is the inhibition rate of pathogen *Fusarium spp*. or *Phytophthora spp*. (%); N_1_ is the number of blank control pathogens; N_2_ is the number of agents treated pathogens.

Levene’s Test for equality of variances was applied to all data on ABM residue concentration, initial cucumber yield and total yield, plant height and total income in greenhouse trials. As the variances between the factor levels were approximately equal (p>0.05), the data were analyzed for significant differences by two-way analysis of variance with factors of ABM rate and fumigants. The data on the inhibition rate of pathogens in the soil of the glasshouses, generated by using various ABM rates combined with fumigants, were analyzed for significant differences by one-way analysis of variance. The *priori* level of significance wasα = 0.05. The Tukey Test was used to determine the differences between treatment means. Statistical analyses were performed using SPSS Statistics V17.0. All the figures were plotted with OriginPro 8.0 software.

## Results and discussion

### Degradation of abamectin in the laboratory

**Fig 1:**
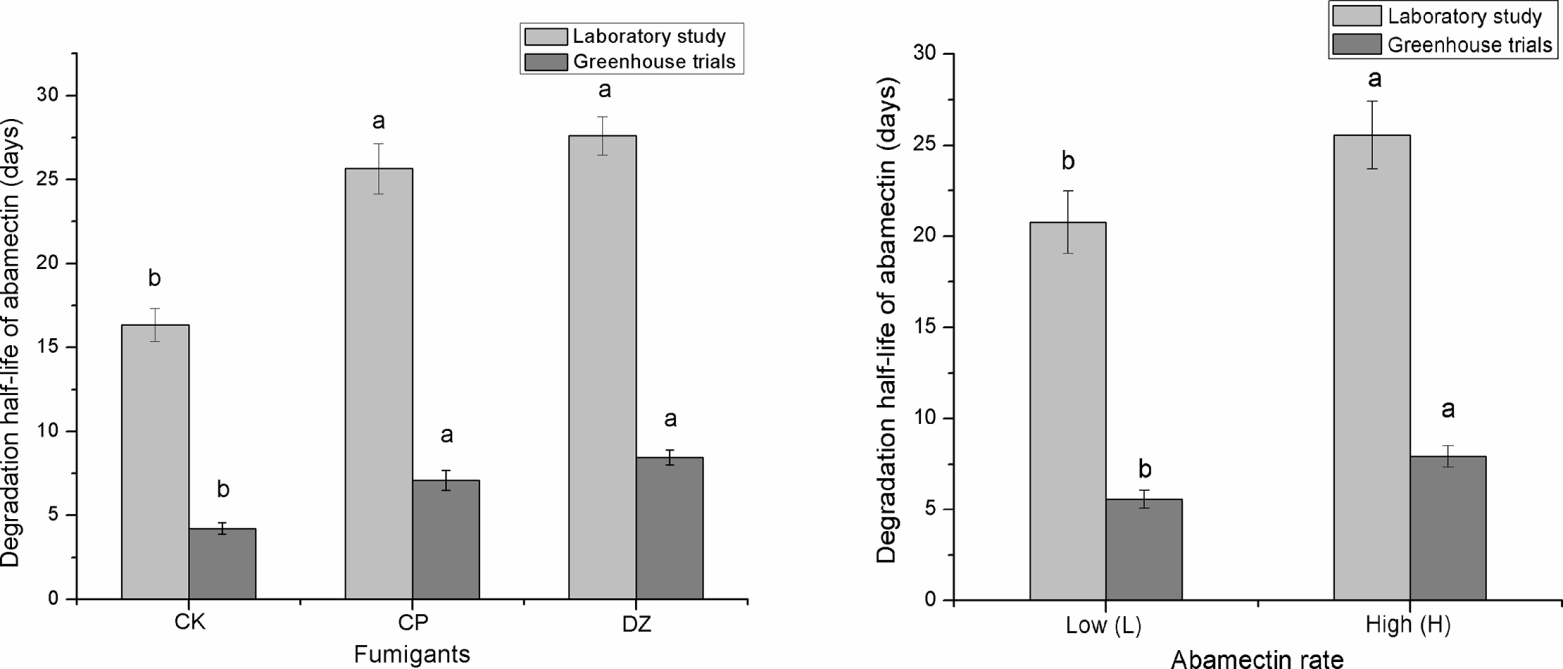
Multiple comparisons of the degradation half-life of abamectin combined with dazomet or chloropicrin in laboratory and greenhouse trials. Different letters (a, b) in the figure indicate significant differences (p < 0.05) according to Tukey test. Error bars indicate the standard error of degradation half-life. The fumigant treatments consisted of six replicate samples in the laboratory and twelve replicate samples in the greenhouse trials. The abamectin treatments consisted of nine replicated samples in the laboratory and eighteen replicate samples in the greenhouse trials.

Linear regression on log-transformed residual data was used to test the goodness of fit of the first-order equation. It described the observed degradation of ABM in the laboratory satisfactorily as the F-value probabilities were all less than 0.05 and the correlation coefficients of linear regression were all greater than 0.85. In addition, the correlation coefficients (R^2^) of the fitted curve were all greater than 0.9 (Table 4). Statistical analysis showed that the two main effects of DZ and CP fumigants (F = 96.02, P = 0.000) and ABM rate (F = 47.19, P = 0.000) had a significant effect on the degradation half-life of ABM, but their interaction (F = 0.57, P = 0.578) was not significant (S1 Table). The results showed that all DZ and CP fumigation treatments significantly (p<0.05) extended the degradation half-life of ABM, compared with the unfumigated control (Fig 1). The average half-life of ABM alone, when used at the low and high dosage rates, was 16.4 days. ABM in combination with DZ or CP produced an average half-life of approximately 27.5 days and 25.6 days, respectively. Moreover, the half-life of ABM in DZ and CP treatments was extended by 1.68 and 1.56 times the untreated control (Table 4). This may be due to the soil fumigants killing most of the soil microorganisms shortly after the treatment commenced including those organisms that degrade ABM [26,38]. ABM is reported to be degraded mainly by microbial organisms [24].

**Table 4:** Degradation rate (k), coefficient of determination (R^2^) and half-life (T^1^/_2_) of abamectin combined with fumigants in the laboratory.

Abamectin rate	Soil fumigant^a^	K	R^2^	T^1^/_2_ (days)
Low (L)	CK	0.0486	0.938	14.3 ± 0.702
	DZ	0.0274	0.973	25.3 ± 0.862
	CP	0.0305	0.971	22.7 ± 0.896
High (H)	CK	0.0377	0.966	18.4 ± 0.603
	DZ	0.0233	0.931	29.7 ± 0.699
	CP	0.0242	0.962	28.6 ± 1.274

^a^DZ, dazomet; CP, chloropicrin; Half-life data are shown as the arithmetic mean with standard error (n = 3).

The different initial concentration of ABM appeared to affect the degradation half-life. The results showed that the degradation of ABM at the high rate (H) was slower than at the low rate (L) (Fig 1). Bull *et al*. (1984) reported the half-life of ABM changed depending on its initial concentration in soil [39]. There were no statistical differences in the ABM degradation rates when ABM was combined with CP or DZ.

The degradation half-life of ABM in the laboratory was consistent with that of previous studies. Lucija *et al*. (2006) reported that the half-life of ABM in sheep dung was about 23 days [40]. Tina *et al*. (2010) reported that the average half-life of ABM in the dung of ABM-treated sheep was about 27 days [41]. Bull *et al*. (1984) found that half-life values ranged from 14 to 56 days for ABM in sand, clay or sandy loam soils under aerobic conditions in the USA [39]. Awasthi *et al*. (2013) reported a much longer half-life for ABM which ranged from 114 to 118 days in N-methyl pyrrolidone solution held at 55°C to 70°C [24]. In an incubation test that examined the degradation dynamics of ABM in soil, Zhang *et al*. (2004) found that the half-life of ABM was 277.3 days in microbe-free conditions and 49.9 days in the presence of microbes, which strongly suggested that the degradation of ABM in soil was mostly by microbes [42].

The degradation of ABM was reported to be much faster in the soil from fields or greenhouses than in the laboratory. Alaa *et al*. (2007) demonstrated that the initial deposit of ABM residues in soils producing dates declined to 66% and 88% of the initial residues 7 and 14 days after ABM application, respectively [43]. Abd-Alrahman *et al*. (2013) observed that the half-life of ABM was 2.38 days in field soil producing cucumbers [21]. When ABM was applied in the greenhouse, Xie *et a*l. (2008) reported the half-life values in soil producing cauliflower ranged from 5.23 to 5.49 days [44]. Under different environmental conditions, the degradation rate of ABM was also different [23, 24]. Degradation of ABM may be influenced by many factors including the type and quantity of micro-organisms in the soil, the soil characteristics, the incidence of sunlight and rain, the soil pH, the temperature and moisture content [45].

### The efficacy of combinations on root-knot nematode in the laboratory

Nematode mortality in the control was less than 6%. All the combinations of ABM / DZ and ABM / CP showed a synergistic effect on RKN control. However, there was no synergistic effect observed on most treatments affecting *Fusarium spp*. and *Phytophthora spp*. The corrected nematode mortality rate of ABM alone at 0.75 mg kg^-1^, DZ alone at 50 mg kg^-1^ and CP alone at 20 mg kg^-1^ were 46.7%, 54.6% and 62.3% respectively (Table 5). The combination of 0.75 mg kg^-1^ ABM and 50 mg kg^-1^ DZ resulted in 75.8% RKN mortality. The combination of 0.75 mg kg^-1^ ABM and 20 mg kg^-1^ CP resulted in 79.9% RKN mortality (Table 5). The corrected nematode mortality rate of ABM in combination with CP and DZ was therefore significantly higher than when ABM, DZ or CP was used alone.

**Table 5:** The efficacy of abamectin, dazomet and chloropicrin on root-knot nematode and soilborne fungi in laboratory studies.

Treatment	Rate (mg kg^-1^)	Corrected mortality rate of root-knot nematode and inhibition rate of soilborne fungi (%)								
		*Meloidogyne spp*.			*Fusarium spp*.			*Phytophthora spp*.		
		Observed value E	Theoretical value E_0_	E-E_0_^a^	Observed value E	Theoretical value E_0_	E-E_0_	Observed value E	Theoretical value E_0_	E-E_0_
Abamectin	0.75	46.7			6.8			13.2		
	1.5	73.1			10.6			18.9		
DZ	50	54.6			71.4			61.7		
	100	78.4			93.8			78.6		
CP	20	62.3			79.6			58.2		
	40	87.6			91.2			85.9		
Abamectin+DZ	0.75+50	77.3	75.8	+2.5	68.2	73.3	-5.1	58.5	66.8	-8.3
	0.75+100	95.2	88.5	+6.7	91.3	94.2	-2.9	83.3	81.4	+1.9
	1.5+50	91.7	87.8	+3.9	64.5	74.4	-9.9	65.7	68.9	-3.2
	1.5+100	100	94.2	+5.8	94.7	94.5	+0.2	82.0	82.6	-0.6
Abamectin+CP	0.75+20	82.2	79.9	+2.3	72.6	81.0	-8.4	60.2	63.7	-3.5
	0.75+40	98.7	93.4	+5.3	87.3	91.8	-4.5	87.6	87.8	-0.2
	1.5+20	92.6	89.9	+2.7	73.0	78.0	-5.0	68.1	66.1	+2.0
	1.5+40	100	96.7	+3.3	95.7	92.1	+3.6	89.5	88.6	+0.9

^a^E- E_0_: If E- E_0_ > 0, the combinations were synergistic and marked as “+”; if E- E_0_ < 0, the combinations were antagonistic and marked as “-”. Data are the means of three replicates.

The combination of 50 mg kg^-1^ DZ or 20 mg kg^-1^ CP with 0.75 mg kg^-1^ ABM did not effectively reduce *Fusarium spp*. and *Phytophthora spp.*. In order to achieve high nematode mortality, and at the same time prevent damage from pathogens such as *Fusarium spp*. and *Phytophthora spp*., the combination of 0.75 mg kg^-1^ ABM and 100 mg kg^-1^ DZ, or 0.75 mg kg^-1^ ABM and 40 mg kg^-1^ CP, could be suitable for application in a greenhouse. These two combinations had a synergistic effect on nematodes, suppressed pathogens and reduced *Meloidogyne spp*., *Fusarium spp*. and *Phytophthora spp*. by 95.2%, 87.3% and 83.3%, respectively (Table 5). Further research is required to find the lowest doses of ABM/DZ and ABM/CP that produce an even higher level of control of these pests in order to minimise the risk of resistance to ABM.

### The effect of CP and DZ on the diffusion rate of abamectin through the epidermis of the root-knot nematode

**Fig 2:**
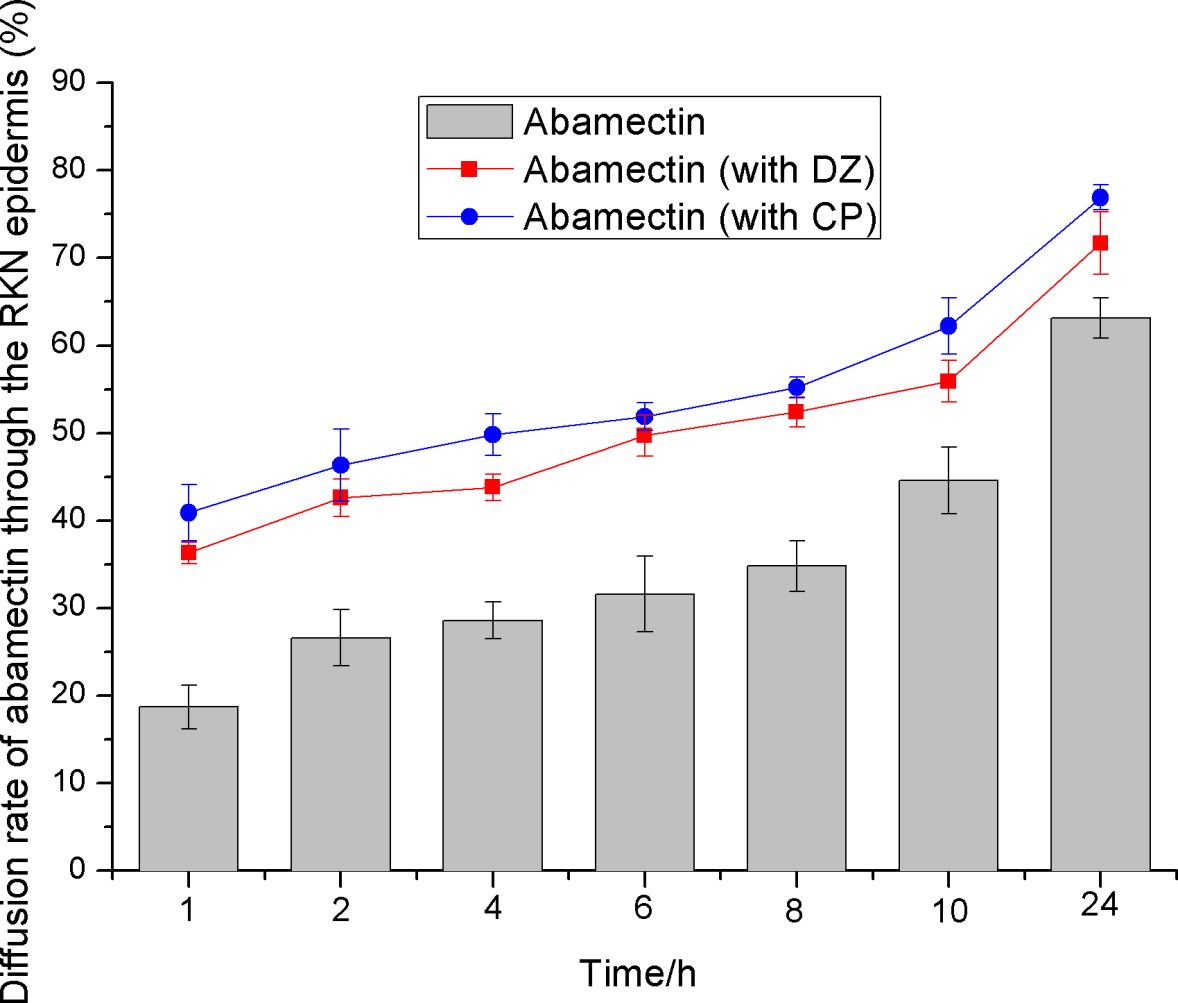
The diffusion rate of abamectin into the epidermis of RKN when abamectin is used alone or on combination with dazomet or chloropicrin. Error bars indicate the standard error of penetration rate between three replicate samples. RKN = Root Knot Nematode.

CP or DZ combined with ABM increased the diffusion rate of ABM through the nematode epidermis, compared to when ABM was used alone (Fig 2). The diffusion rate of ABM in 1 h (with DZ: 36.3%, with CP: 40.9%) was more than double the diffusion rate of ABM used alone for 1 h (18.7%) (Table 6). CP facilitated the diffusion of ABM more than DZ. Apart from this combination, the effect of ABM on the diffusion rate of CP or DZ also increased slightly, but the diffusion effect of these fumigants on ABM is greater than ABM on them. CP or DZ may have interacted with ABM in the process of diffusing through the nematode epidermis, while also simultaneously facilitating ABM’s diffusion of the nematode epidermis.

**Table 6:** The percentage diffusion rate into the nematode epidermis by abamectin combined with dazomet or chloropicrin used alone or in combination.

Treatment		Hours						
		1	2	4	6	8	10	24
Single	Abamectin	18.7^a^	26.6	28.6	31.6	34.8	44.6	63.1
	DZ	36.3	47.3	55.5	67.3	78.8	84.5	100
	CP	43.8	54.5	66.2	78.3	89.1	100	100
Combination	Abamectin (with DZ)	36.3	42.6	43.8	49.7	54.4	55.9	71.7
	Abamectin (with CP)	40.9	46.3	49.8	51.9	54.7	62.2	76.9
	DZ	42.3	51.2	64.1	76.2	82.5	87.8	100
	CP	48.2	59.1	68.9	82.3	93.7	100	100

^a^18.7: penetration rates, the unit is %.

Pesticides can have an impact only when they pass through a plant and make direct contact with the target pest. The efficacy of a pesticide is not only a function of its toxicity, but also its ability to diffuse across the pest epidermis. Many pesticides inadvertently act in ways that fortify the epidermis of pests, which reduces pesticide efficacy and increases the risk of pest resistance [46,47].

Fumigants may have the ability to work together to penetrate biological cells. For example, two fumigants may have a synergistic effect when fumigant A increases the permeability of the cell wall, making it easier for fumigant B to follow and diffuse into the cell [48]. ABM is a new type of nerve agent that is reported to interact with biochemical target sites γ-aminobutyric acid (GABA) in the nematode nervous system and the glutamate-controlled Cl^-^ channel, leading eventually to nematode paralysis and death [49,50]. The combination of ABM and fumigant may act synergistically by improving the ability of ABM to penetrate the nematode epidermis, thereby reducing the time for ABM to reach these target sites.

### Degradation of nematicide in greenhouse trials

The degradation of ABM was also monitored in the cucumber greenhouse for a total of 90 d. Levene’s Test showed that the variances were equal across the degradation data obtained from Trials 1 and 2 (F = 3.325, p = 0.077). The T-test for equality of means showed that the degradation half-life of ABM in Trials 1 and 2 were not significantly different (t = 0.539, p = 0.593; S2 Table). Therefore, all the degradation data were combined for a two-way analysis of variance with factors of ABM rate and fumigant type. Statistical analysis showed that fumigants (F = 27.91, p = 0.000) and ABM concentration (F = 12.52, p = 0.001) had a significant effect on the degradation half-life of ABM, while their interaction (F = 0.79, p = 0.460) was not significant (S3 Table). The lack of statistical significance may have been due to slight differences in soil characteristics between the two trials, differences in soil microbial species and quantity, or field conditions that differed slightly between treatments. The degradation half-life of ABM showed significant differences (p<0.05) between the fumigated and unfumigated soil treatments. All the treatments that used DZ and CP extended the degradation half-life of ABM (Fig 1). The average half-life of the unfumigated treatment control in the two trials was about 4.2 days, while DZ and CP fumigant treatments approximately doubled the half-life values of ABM to 8.5 and 7.1 days, respectively. The half-life values of ABM when combined with DZ or CP were extended 2.02 and 1.69 times more than the control half-life (Table 7). The results also showed that the degradation rate of ABM in the greenhouse trials using a high initial dose (H) was significance slower than when a low initial dose (L) was used (Fig 1).

**Table 7:** Degradation rate (k), coefficient of determination (R^2^) and half-life (T^1^/_2_) of abamectin combined with dazomet or chloropicrin in greenhouse trials.

Trials site	Abamectin rate	Soil fumigant	K	R^2^	T^1^/_2_ (days)
Trial 1	Low (L)	CK	0.1583	0.961	4.4 ±0.404
		DZ	0.1055	0.969	6.5 ±0.603
		CP	0.0954	0.913	7.3 ±0.557
	High (H)	CK	0.1319	0.925	5.3 ±0.556
		DZ	0.0793	0.900	8.7 ±0.794
		CP	0.0809	0.936	8.6 ±0.493
Trial 2	Low (L)	CK	0.2459	0.873	2.9 ±0.600
		DZ	0.0872	0.943	7.9 ±0.851
		CP	0.1255	0.989	5.5 ±0.794
	High (H)	CK	0.1609	0.959	4.3 ±0.400
		DZ	0.0645	0.975	10.7 ±1.301
		CP	0.1009	0.951	6.9 ±0.755

Half-life data are shown as the arithmetic mean with standard error (n = 3).

The degradation half-life of ABM in the greenhouse trials was significantly faster than that in the laboratory. Faster degradation in the greenhouse may have been due to the prevalence of conditions in the field, such as rainfall, light, high temperature and other factors that have been reported to degrade ABM [24]. The soil microbial population may also have been larger in the greenhouse than in the laboratory [51,52]. Fumigant treatments significantly prolonged the time for ABM degradation than in the unfumigated treatments in the greenhouse and in the laboratory. Prolongation of the time for ABM degradation may be due to fumigants destroying most of the soil microbial community. Microbes were reported to degrade ABM [26,38]. Microbe degradation by CP and DZ enabled larger concentrations of ABM to interact with nematodes in the field over a prolonged period of time, which could have contributed to a synergistic effect when ABM and these fumigants were combined.

### Effect on the root-knot nematode, soilborne fungi and cucumber growth

The soil from Trials 1 and 2 had been heavily infested by RKN for many years. The initial population number of RKN in these soils averaged 260/100 g and 430/100 g before fumigation in Trials 1 and 2, respectively. The initial number of *Fusarium spp*. and *Phytophthora spp*. colonies in the soil averaged 6172/g and 7516/g in Trial 1, and 5340/g and 6188/g in Trial 2, respectively. The corrected mortality rate of RKN and the inhibition rate of soilborne fungi in the untreated control were significantly different when DZ or CP was combined with ABM. On the 7th day after ABM application, the nematode mortality was higher in the combinations than when ABM, DZ or CP was used alone. DZ in combination with ABM performed better as a nematicide than when CP was combined with ABM. As expected, the nematode mortality and fungal control gradually decreased with increased time after the initial treatment application. Ninety days after application, nematode control using CP/ABM or DZ/ABM, or CP or DZ used alone, was significantly greater than ABM alone. Recovery of the nematode population when ABM was used alone suggested that its presence in soil was rather transitory. All the combination treatments reduced RKN by at least 82.5% 7 days after the initial applications in Trials 1 and 2. ABM combined with DZ or CP reduced nematodes by 92.1% and 90.5%, respectively (Table 8). In the first 35 days, the nematode and fungal control when ABM was combined with DZ or CP were significantly different than when each was used alone, while there was no different between combination treatments of low (L) or high (H) ABM concentrations. CP and CP/ABM treatments inhibited *Fusarium spp*. significantly more than DZ and DZ/ABM, but there was no difference on *Phytophthora spp*. CP or DZ used alone caused higher mortality of *Fusarium spp*. than *Phytophthora spp*. than ABM. The effect of ABM on fungi was not effective, and even the combination of ABM and fumigant had no synergistic effect on fungi. Although the trials showed that ABM gave 15.3% to 43.7% inhibition of *Fusarium spp*. than *Phytophthora spp* 7–90 days after its initial application (Table 8), this fungicidal effect might have been due to solar radiation as a result of the use of black polyethylene film that raised the temperature of the soil [53]. The combination of DZ or CP with ABM at a low dose prolonged the effective control of RKN from an application every year to an application every two years, which offers significant practical and cost-saving benefits to farmers.

**Table 8:** The efficacy of abamectin and fumigants on root-knot nematode and soilborne fungi in greenhouse trials.

Trials site	Soil treatments	Reagent rate (g m^-2^)	Corrected mortality rate of root-knot nematode and inhibition rate of soilborne fungi (%)										
			*Meloidogyne spp*.					*Fusarium spp*.			*Phytophthora spp*.		
			7d^a^	21d	35d	60d	90d	7d	35d	90d	7d	35d	90d
Trial 1	CK^b^	--	0e^c^	0d	0e	0e	0d	0d	0f	0e	0d	0d	0e
	ABM	0.375	68.2d	75.0c	50.1d	43.1d	28.6c	35.1c	28.3d	27.4d	34.0c	22.3c	20.8d
	ABM	0.75	84.1b	82.5b	67.3c	52.7c	33.4c	31.2c	19.2e	32.4d	32.5c	15.3c	22.3d
	DZ	30	82.5b	77.7c	71.8b	65.8b	52.7bc	79.2b	62.5c	55.3c	89.7a	67.2ab	43.7c
	ABM+DZ	0.375+30	92.1a	90.3ab	85.4a	76.3a	81.6a	75.7b	64.5c	51.2c	89.0a	62.3b	56.9b
	ABM+DZ	0.75+30	97.6a	94.9a	89.1a	81.6a	69.4b	73.5b	72.3b	57.2c	84.9a	73.2a	65.8ab
	CP	20	76.2c	82.5b	74.5b	60.5b	52.7bc	95.6a	98.3a	86.7ab	73.2b	84.0a	78.5a
	ABM+CP	0.375+20	95.2a	97.6a	87.2a	73.6ab	65.6b	92.0a	92.4a	82.5b	80.8ab	82.3a	72.0a
	ABM+CP	0.75+20	90.5ab	91.6a	92.8a	78.9a	61.1b	97.9a	96.0a	93.5a	79.6ab	76.6a	64.7ab
Trial 2	CK	--	0d	0c	0d	0f	0d	0d	0d	0e	0c	0d	0e
	ABM	0.375	62.3c	72.6b	43.3c	37.1e	37.5bc	42.6c	38.3c	23.7d	42.3b	35.7c	42.3c
	ABM	0.75	67.8c	78.6ab	62.5b	45.7d	27.7c	43.7c	34.3c	27.6d	35.6b	32.8c	23.6d
	DZ	30	89.2b	82.4ab	73.1ab	51.4c	45.8b	86.7b	73.2b	62.3b	92.4a	83.2ab	37.1c
	ABM+DZ	0.375+30	100a	74.7b	81.7a	71.5b	62.5a	92.4a	76.8b	54.9c	93.2a	81.7b	56.3bc
	ABM+DZ	0.75+30	94.6a	88.3a	82.5a	68.6b	70.6a	89.6ab	83.4ab	55.6bc	87.5a	78.2b	61.2b
	CP	20	83.9bc	84.0a	79.3a	65.7bc	41.7bc	98.2a	92.3a	84.5ab	90.4a	82.3bb	85.6a
	ABM+CP	0.375+20	96.2a	87.5a	85.6a	85.7a	58.4ab	97.5a	95.6a	86.7a	93.0a	95.4a	87.0a
	ABM+CP	0.75+20	98.2a	92.3a	86.9a	74.3b	66.7a	99.6a	93.2a	92.4a	95.5a	91.8a	85.9a

7d^a^: “d” in this line represents “days” and refers to the time after the application of abamectin combined with fumigants or used alone

CK^b^: CK = untreated control (unfumigated and without ABM); the corrected mortality rate and inhibition rate of CK were specified as “0”.

0e^c^: Data are the means of three replicates. Different letters (a-f) in the same column in each trial indicate significant differences (p < 0.05) according to Tukey test. The unit of corrected mortality rate and inhibition rate were percentage.

#### Effect on the growth of cucumber and total farmer income

**Fig 3:**
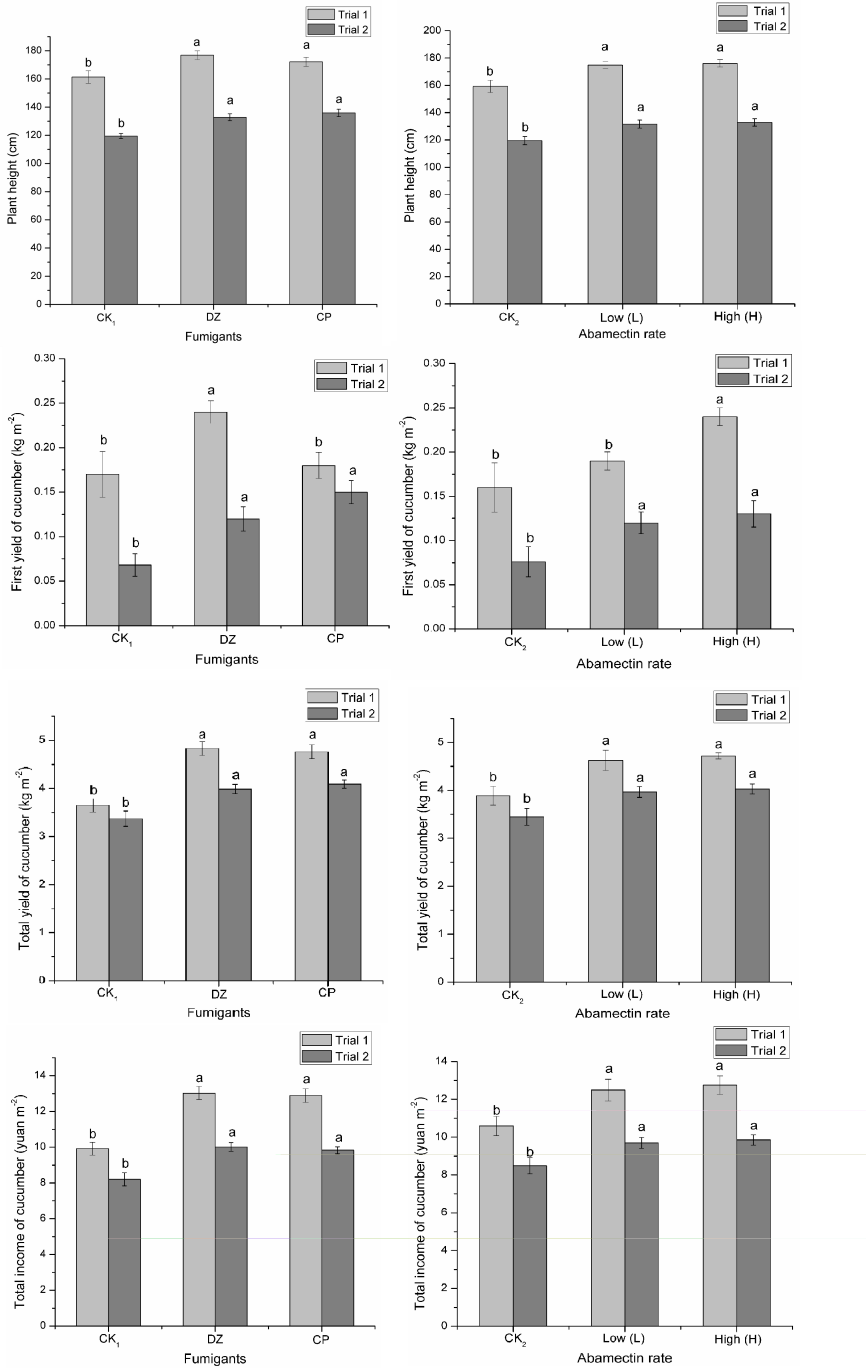
Multiple comparisons of cucumber growth and total income when abamectin is combined with dazomet or chloropicrin in greenhouse trials. CK1 is the control without dazomet or chloropicrin; CK2 is the control without abamectin. Different letters (a, b) in the same column in each trial indicate significant differences (p < 0.05) according to Tukey test. Error bars indicate the standard error between nine replicates in plant height (each plant height treatment was measured from 20 cucumber plants), first yield, total yield and total income.

**Fig 4:**
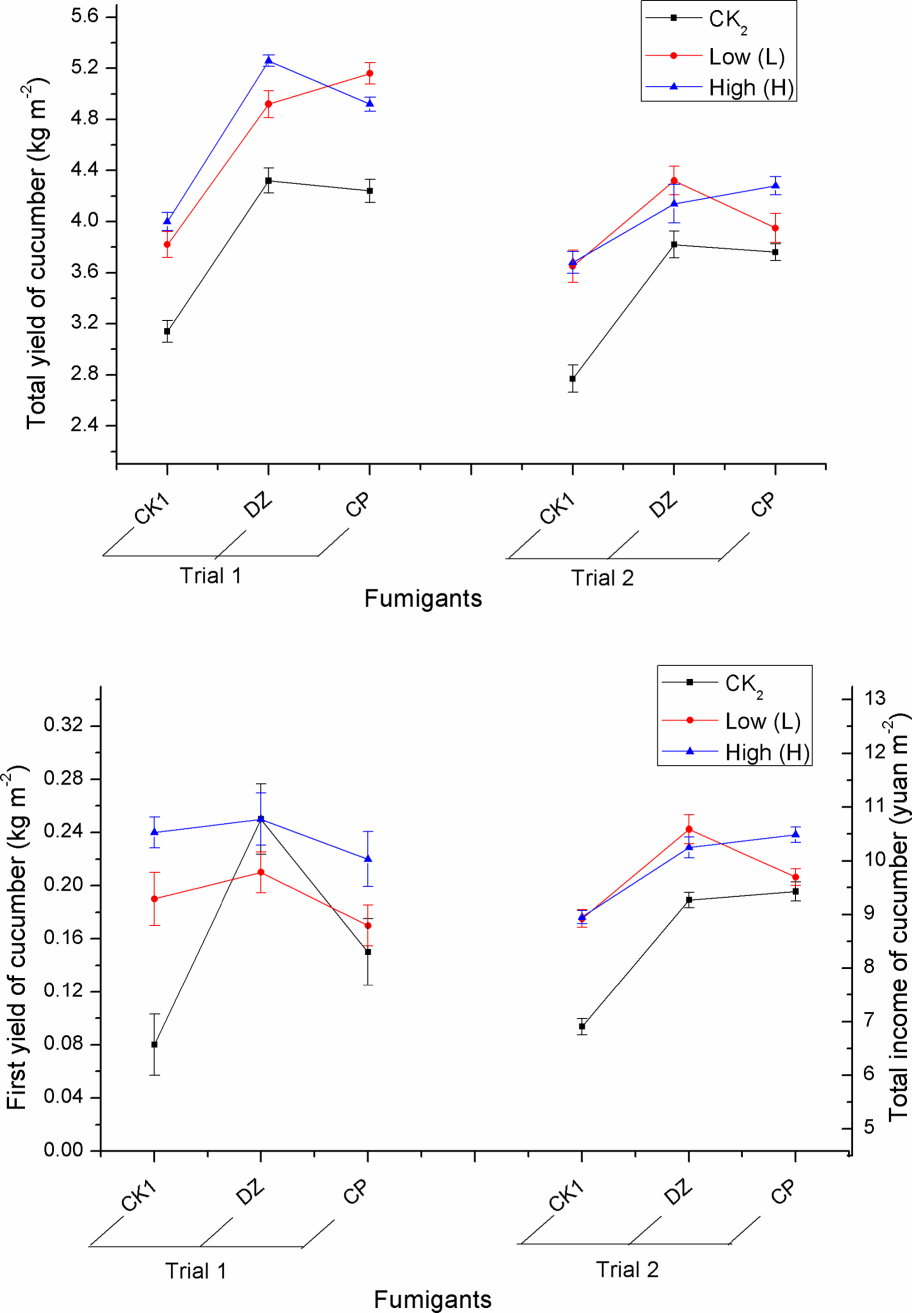
The effect of abamectin combined with fumigants on total yield, initial yield and total income in a greenhouse study. Error bars indicate the standard error of total yield, first yield and total income between three replicate samples.

Cucumber plant height, total yield and total income in Trial 1 were higher than in Trial 2 (Fig 3). This may be attributed to differences in fertilizer application and crop management between the two greenhouses. The main effects of ABM rate and fumigants on cucumber plant height, initial yield, total yield and total income were significantly different, but their interaction was only significantly different on total yield (Trial 1: F = 3.24, p = 0.036; Trial 2: F = 3.98, p = 0.018), initial yield (F = 4.80, p = 0.008) in Trial 1, and total income (F = 3.58, p = 0.026) in Trial 2 (S4 Table). The results showed that cucumber plant height, initial yield in Trial 2, and total income in Trial 1 in the fumigation treatments, were significantly higher than that of unfumigated treatment (Fig 3). In the unfumigated treatments this might have been due to the heavy infestation of RKN, cucumber fusarium wilt, cucumber blight, as well as cucumber root rot and weeds, resulting in seedling mortality and reduced yield. Moreover, the cucumber plant height, initial yield in Trial 2 and total income in Trial 1 using ABM at low or high rates were significantly higher than for a crop produced without the use of ABM (Fig 3). The total yield of cucumbers following a DZ or CP treatment was significantly greater than in the unfumigated treatment when the ABM concentration was low, high or not applied at all in Trials 1 and 2. However, when DZ or CP was combined with ABM, the total crop yield was not significantly different when ABM was used at the low or high rate (Fig 4 and S5 Table). Therefore a low rate of ABM combined with CP or DZ was both more economical and reasonable for cucumber production in a greenhouse. When low or high rates of ABM were used in Trial 1, the first yield of cucumbers in the DZ treatment was greater than in the CP treatment, but there were no significant different except for CK_2_ treatment (F = 8.82, p = 0.002). A low rate of ABM in Trial 2 resulted in the total income in the DZ treatment being significantly (F = 3.63, p = 0.045) greater than the total yield in the CP treatment (Fig 4 and S6 Table). DZ/ABM’s better performance in this research suggests it should be selected in preference to CP/ABM in order to improve the first cucumber yield and total crop income.

## Conclusion

DZ and CP combined with ABM significantly extended the degradation half-life of ABM in this research conducted on the soil sampled from greenhouses and carried out in the laboratory. ABM degradation rate in the soil was slower when a high rather than low initial dose of ABM was used. Laboratory experiments showed that all DZ/ABM or CP/ABM combinations controlled RKN through a synergistic effect. The synergistic mechanism may have been due an increased presence of ABM as a result of the fumigants DZ and CP eliminating most of the soil microbes including the bacteria known to degrade ABM. This improved the short term efficacy of ABM as a nematicide and doubled the longer term effective control period from one to two years. The laboratory study showed that ABM diffused through the nematode epidermis more quickly when ABM was combined with CP or DZ than when ABM was used alone. The better performance of low dose of ABM combined with DZ suggested this combination would be an economic and practical way to control nematode and soilborne fungi in a greenhouse producing cucumbers.

## Supporting information

S1 Table Test of between-subject effects of half-life in fumigants and abamectin rate in the laboratory. (DOC)

S2 Table Independent sample t-test of the degradation half-life of ABM in Trial 1 and Trial 2. (DOCX)

S3 Table Test of between-subjects effects of half-life of abamectin combined with fumgiants in greenhouse trials. (DOCX)

S4 Table Test of between-subjects effects of cucumber plant growth, crop yield (initial and total) and income when abamectin was combined with different fumigants or used alone in greenhouse trials. (DOCX)

S5 Table Tests of significance for total yield in greenhouse trials using UNIQUE sums of squares. (DOCX)

S6 Table Tests of significance for first yield in trial 1 and total income in trial 2 using UNIQUE sums of squares. (DOCX)
