# Supplementary material for: Effect of soil fumigants on degradation of abamectin and their combination synergistic effect to root-knot nematode
Source: PLoS One. 2018 Jun 11;13(6):e0188245. doi: 10.1371/journal.pone.0188245 (PMC5995350; doi:10.1371/journal.pone.0188245)
Supplement: S1 Table — (DOC) [file pone.0188245.s001.doc]

**S1 Table.** Test of between-subject effects of half-life in fumigants and abamectin rate in the laboratory.

| Source of Variation | SS^a^ | DF^b^ | MS ^c^ | F | P |
| --- | --- | --- | --- | --- | --- |
| Residuals | 27.1 | 12 | 2.26 |  |  |
| Nematicide rate | 106.6 | 1 | 106.58 | 47.19*** | 0.000 |
| Fumigant | 433.7 | 2 | 216.86 | 96.02*** | 0.000 |
| Nematicide rate BY Fumigant | 2.6 | 2 | 1.29 | 0.57 | 0.578 |
| (Model) | 542.8 | 5 | 108.57 | 48.08*** | 0.000 |
| (Total) | 569.9 | 17 |  |  |  |

^a^SS= Sum of squares, ^b^DF= Degrees of freedom，^c^MS= Mean square. The significance level of the F values.(* for p<0.05, ** for p<0.01, and *** for p<0.001)
