# Supplementary material for: Effect of soil fumigants on degradation of abamectin and their combination synergistic effect to root-knot nematode
Source: PLoS One. 2018 Jun 11;13(6):e0188245. doi: 10.1371/journal.pone.0188245 (PMC5995350; doi:10.1371/journal.pone.0188245)
Supplement: S2 Table — (DOCX) [file pone.0188245.s002.docx]

**S2 Table.** Independent sample t-test of the degradation half-life of ABM in Trial 1 and Trial 2

|  | | Levene Test for Equal variances assumed | | t-test for Equality of Means | | | | | | |
| --- | --- | --- | --- | --- | --- | --- | --- | --- | --- | --- |
|  |  | F | Sig. | t | df | Sig.(2-tailed) | Mean Difference | Std. Error Difference | 95% Confidence | |
|  |  |  |  |  |  |  |  |  | Lower | Upper |
| VAR00002 | Equal variances assumed | 3.325 | 0.077 | 0.539 | 34 | 0.593 | 0.4333 | 0.80412 | -1.20083 | 2.06749 |
|  | Equal variances not assumed |  |  | 0.539 | 28.964 | 0.594 | 0.4333 | 0.80412 | -1.21135 | 2.07802 |
