# Supplementary material for: Effect of soil fumigants on degradation of abamectin and their combination synergistic effect to root-knot nematode
Source: PLoS One. 2018 Jun 11;13(6):e0188245. doi: 10.1371/journal.pone.0188245 (PMC5995350; doi:10.1371/journal.pone.0188245)
Supplement: S3 Table — (DOCX) [file pone.0188245.s003.docx]

**S3 Table.** Test of between-subjects effects of half-life of abamectin combined with fumgiants in greenhouse trials.

| Source of Variation | SS | DF | MS | F | P |
| --- | --- | --- | --- | --- | --- |
| Residuals | 59.9 | 30 | 1.99 |  |  |
| Nematicide rate | 25.0 | 1 | 25.00 | 12.52** | 0.001 |
| Fumigant | 111.5 | 2 | 55.73 | 27.91*** | 0.000 |
| Nematicide rate BY Fumigant | 3.2 | 2 | 1.59 | 0.79 | 0.460 |
| (Model) | 139.6 | 5 | 27.92 | 13.98*** | 0.000 |
| (Total) | 199.6 | 35 |  |  |  |

^a^SS= Sum of squares, ^b^DF= Degrees of freedom，^c^MS= Mean square. The significance level of the F values.(* for p<0.05, ** for p<0.01, and *** for p<0.001)
