# Supplementary material for: Effect of soil fumigants on degradation of abamectin and their combination synergistic effect to root-knot nematode
Source: PLoS One. 2018 Jun 11;13(6):e0188245. doi: 10.1371/journal.pone.0188245 (PMC5995350; doi:10.1371/journal.pone.0188245)
Supplement: S4 Table — (DOCX) [file pone.0188245.s004.docx]

**S4 Table.** Test of between-subjects effects of cucumber plant growth, crop yield (initial and total) and income when abamectin was combined with different fumigants or used alone in greenhouse trials.

| Trials | Independent variable | Source of Variation | SS | DF | MS | F | P |
| --- | --- | --- | --- | --- | --- | --- | --- |
| Trial 1 | Plant height | Nematicide rate | 1548.9 | 2 | 774.44 | 12.45^***^ | 0.000 |
|  |  | Fumigant | 1135.7 | 2 | 567.86 | 9.13^**^ | 0.002 |
|  |  | Nematicide rate BY Fumigant | 219.2 | 4 | 54.79 | 0.88 | 0.495 |
|  |  | (Model) | 2903.8 | 8 | 362.9 | 5.83^**^ | 0.001 |
|  | Initial yield | Nematicide rate | 0.03 | 2 | 0.013 | 10.61^**^ | 0.001 |
|  |  | Fumigant | 0.02 | 2 | 0.012 | 9.18^**^ | 0.002 |
|  |  | Nematicide rate BY Fumigant | 0.01 | 4 | 0.001 | 4.80^**^ | 0.008 |
|  |  | (Model) | 0.07 | 8 | 0.01 | 7.35^***^ | 0.000 |
|  | Total yield | Nematicide rate | 3.8 | 2 | 1.88 | 89.25^***^ | 0.000 |
|  |  | Fumigant | 7.9 | 2 | 3.96 | 188.11^***^ | 0.000 |
|  |  | Nematicide rate BY Fumigant | 0.3 | 4 | 0.07 | 3.24^*^ | 0.036 |
|  |  | (Model) | 3.8 | 2 | 1.88 | 89.25^***^ | 0.000 |
|  | Total income | Nematicide rate | 24.9 | 2 | 12.44 | 80.81^***^ | 0.000 |
|  |  | Fumigant | 55.7 | 2 | 27.85 | 180.92^***^ | 0.000 |
|  |  | Nematicide rate BY Fumigant | 1.61 | 4 | 0.40 | 2.61 | 0.070 |
|  |  | (Model) | 82.18 | 8 | 1027 | 66.74^***^ | 0.000 |
| Trial 2 | Plant height | Nematicide rate | 455.6 | 2 | 227.53 | 14.08^***^ | 0.000 |
|  |  | Fumigant | 1366.4 | 2 | 683.19 | 42.29^***^ | 0.000 |
|  |  | Nematicide rate BY Fumigant | 141.2 | 4 | 35.29 | 2.18 | 0.112 |
|  |  | (Model) | 1962.6 | 8 | 245.32 | 15.19^***^ | 0.000 |
|  | Initial yield | Nematicide rate | 0.02 | 2 | 0.009 | 10.12^**^ | 0.001 |
|  |  | Fumigant | 0.03 | 2 | 0.014 | 16.33^***^ | 0.000 |
|  |  | Nematicide rate BY Fumigant | 0.01 | 4 | 0.001 | 1.31 | 0.302 |
|  |  | (Model) | 0.05 | 8 | 0.006 | 7.27^***^ | 0.000 |
|  | Total yield | Nematicide rate | 1.9 | 2 | 0.93 | 26.77^***^ | 0.000 |
|  |  | Fumigant | 2.8 | 2 | 1.40 | 40.49^***^ | 0.000 |
|  |  | Nematicide rate BY Fumigant | 0.6 | 4 | 0.14 | 3.98^*^ | 0.018 |
|  |  | (Model) | 5.2 | 8 | 0.65 | 18.80^***^ | 0.000 |
|  | Total income | Nematicide rate | 10.1 | 2 | 5.03 | 24.17^***^ | 0.000 |
|  |  | Fumigant | 17.8 | 2 | 8.88 | 42.68^***^ | 0.000 |
|  |  | Nematicide rate BY Fumigant | 2.9 | 4 | 0.74 | 3.58^*^ | 0.026 |
|  |  | (Model) | 30.8 | 8 | 3.85 | 18.5^***^ | 0.000 |

^a^SS= Sum of squares, ^b^DF= Degrees of freedom，^c^MS= Mean square. The significance level of the F values.(* for p<0.05, ** for p<0.01, and *** for p<0.001)
