# Supplementary material for: Effect of soil fumigants on degradation of abamectin and their combination synergistic effect to root-knot nematode
Source: PLoS One. 2018 Jun 11;13(6):e0188245. doi: 10.1371/journal.pone.0188245 (PMC5995350; doi:10.1371/journal.pone.0188245)
Supplement: S5 Table — (DOCX) [file pone.0188245.s005.docx]

**S5 Table.** Tests of significance for total yield in greenhouse trials using UNIQUE sums of squares

| Trials | Source of Variation | SS | DF | MS | F | P |
| --- | --- | --- | --- | --- | --- | --- |
| Trial 1 | Fumigants WITHIN CK_2_ | 2.57 | 2 | 1.28 | 6.22** | 0.008 |
|  | Fumigants WITHIN Low rate (L) | 2.55 | 2 | 1.27 | 6.17** | 0.008 |
|  | Fumigants WITHIN High rate (H) | 3.06 | 2 | 1.53 | 7.42** | 0.004 |
|  | WITHIN+RESIDUAL （1） | 4.13 | 20 | 0.21 |  |  |
|  | Nematicide rate WITHIN CK_1_ | 1.23 | 2 | 0.62 | 1.49 | 0.249 |
|  | Nematicide rate WITHIN CP | 1.43 | 2 | 0.72 | 1.73 | 0.203 |
|  | Nematicide rate WITHIN DZ | 1.36 | 2 | 0.68 | 1.64 | 0.219 |
|  | WITHIN+RESIDUAL （2） | 8.29 | 20 | 0.41 |  |  |
| Trial 2 | Fumigants WITHIN CK_2_ | 2.09 | 2 | 1.04 | 8.42** | 0.002 |
|  | Fumigants WITHIN Low rate (L) | 0.86 | 2 | 0.43 | 3.92* | 0.037 |
|  | Fumigants WITHIN High rate (H) | 0.59 | 2 | 0.30 | 2.39 | 0.118 |
|  | WITHIN+RESIDUAL （1） | 2.48 | 20 | 0.12 |  |  |
|  | Nematicide rate WITHIN CK_1_ | 1.60 | 2 | 0.80 | 4.68* | 0.022 |
|  | Nematicide rate WITHIN CP | 0.42 | 2 | 0.21 | 1.21 | 0.318 |
|  | Nematicide rate WITHIN DZ | 0.38 | 2 | 0.19 | 1.12 | 0.345 |
|  | WITHIN+RESIDUAL （2） | 3.43 | 20 | 0.17 |  |  |
