# Supplementary material for: Effect of soil fumigants on degradation of abamectin and their combination synergistic effect to root-knot nematode
Source: PLoS One. 2018 Jun 11;13(6):e0188245. doi: 10.1371/journal.pone.0188245 (PMC5995350; doi:10.1371/journal.pone.0188245)
Supplement: S6 Table — (DOCX) [file pone.0188245.s006.docx]

**S6 Table.** Tests of significance for first yield in trial 1 and total income in trial 2 using UNIQUE sums of squares

| Trials | Source of Variation | SS | DF | MS | F | P |
| --- | --- | --- | --- | --- | --- | --- |
| First yield in Trial 1 | Fumigants WITHIN CK_2_ | 0.04 | 2 | 0.02 | 8.82** | 0.002 |
|  | Fumigants WITHIN Low rate (L) | 0.00 | 2 | 0.00 | 0.48 | 0.624 |
|  | Fumigants WITHIN High rate (H) | 0.00 | 2 | 0.00 | 0.28 | 0.757 |
|  | WITHIN+RESIDUAL （1） | 0.05 | 20 | 0.00 |  |  |
|  | Nematicide rate WITHIN CK1 | 0.04 | 2 | 0.02 | 8.73** | 0.002 |
|  | Nematicide rate WITHIN CP | 0.01 | 2 | 0.00 | 1.69 | 0.209 |
|  | Nematicide rate WITHIN DZ | 0.00 | 2 | 0.00 | 0.69 | 0.511 |
|  | WITHIN+RESIDUAL （2） | 0.04 | 20 | 0.00 |  |  |
| Total income in Trial 2 | Fumigants WITHIN CK2 | 12.28 | 2 | 6.14 | 8.89** | 0.002 |
|  | Fumigants WITHIN Low rate (L) | 4.96 | 2 | 2.44 | 3.63* | 0.045 |
|  | Fumigants WITHIN High rate (H) | 4.19 | 2 | 2.10 | 3.03 | 0.071 |
|  | WITHIN+RESIDUAL （1） | 13.81 | 20 | 0.69 |  |  |
|  | Nematicide rate WITHIN CK1 | 8.39 | 2 | 4.20 | 3.90* | 0.037 |
|  | Nematicide rate WITHIN CP | 1.86 | 2 | 0.93 | 0.86 | 0.437 |
|  | Nematicide rate WITHIN DZ | 2.79 | 2 | 1.39 | 1.30 | 0.296 |
|  | WITHIN+RESIDUAL （2） | 21.51 | 20 | 1.08 |  |  |
